# ‘Communication Is Crucial’: A Qualitative Study of Patient Expectations of Diagnostic Tests in Emergency Medicine Practice

**DOI:** 10.1111/hex.70648

**Published:** 2026-03-26

**Authors:** Vinay Gangathimmaiah, Rebecca Evans, Tarun Sen Gupta, Karen Cupitt, Sharon Oakley, Karen Carlisle

**Affiliations:** ^1^ Department of Emergency Medicine Townsville University Hospital Townsville Australia; ^2^ College of Medicine and Dentistry James Cook University Townsville Australia; ^3^ Community Collaborator Townsville University Hospital Townsville Australia

**Keywords:** diagnosis, emergency, expectation, Patient, test

## Abstract

**Introduction:**

Value‐based diagnostic tests improve patient safety. Understanding patient expectations can enhance the value of diagnostic tests in Emergency Departments. The aim of this study was to explore patient expectations about diagnostic tests to inform diagnostic stewardship in emergency medicine practice.

**Methods:**

A qualitative study of adult patients who had received care at Townsville University Hospital Emergency Department was conducted. The study was promoted through e‐mail and social media. A convenience sample of consenting participants was recruited until data adequacy was achieved. Data were collected using focus groups with a pilot‐tested guide to ensure clarity and consistency. Data were analysed by lead author using inductive content analysis to generate codes and themes. Themes were validated for credibility, transferability, dependability and confirmability using the following trustworthiness criteria: reflexivity, rich description, audit trail and participant validation.

**Results:**

Five focus groups were conducted with 22 participants. Therapeutic relationship, communication, patient‐centred care and quality of care were identified as the major themes of patient expectations when seeking Emergency Department care. Diagnostic test deliberations were expected to be respectful, trustworthy, empathetic and culturally safe. Patient engagement in decision making about diagnostic tests was considered essential to address individual symptoms and concerns. Participants expressed varied expectations regarding discussion about rationale and risks of tests. Questioning clinicians about tests was perceived to be a challenge due to power imbalance. Participants disagreed with defensive tests performed to minimise medicolegal liability. Timeliness of tests was acknowledged as resource dependent. Clinician‐patient conflicts about tests were thought to be best resolved through a collaborative approach.

**Conclusions:**

Patients have diverse and nuanced expectations of diagnostic tests in emergency departments. Patients trust emergency clinicians to listen empathetically, explain carefully and choose tests wisely. Patient values, preferences, life experiences and vulnerability should be factored into decision‐making about tests. Clinician‐patient partnerships grounded in effective communication are essential for diagnostic stewardship.

**Patient or Public Contribution:**

Two patients with lived experience were involved in study design, data collection and review of final manuscript.

**Abbreviations**
CT: computed tomography
ED: emergency department
GP: general practitioner
ILO: Indigenous Liaison Officer

## 1 Introduction

Value‐based diagnostic tests enhance scientific, efficient, effective and equitable use of resources to benefit patients [1]. Low‐value diagnostic tests can harm patients and health care systems [2, 3]. and account for a significant proportion of Emergency Department (ED) tests [3, 4]. The reported prevalence of ED low‐value tests ranges from 30% of imaging tests(chest radiographs, cranial CT) in a multisite study across all Canadian EDs [4] to 50% of laboratory tests(urine cultures, coagulation studies) in a single‐site Australian ED [3]. De‐implementation of low‐value diagnostic tests can augment patient safety and resource sustainability [5].

Diagnostic stewardship, the right test for the right patient at the right time, can enable emergency clinicians to de‐implement low‐value tests [6]. Stewardship improves patient care and outcomes by optimal use of diagnostic tests whilst ensuring sustainable resource use [6]. Optimal testing minimises diagnostic error and reduces patient harm from wrong, delayed or missed diagnoses [7].

Diagnostic stewardship requires emergency clinicians to navigate underlying causes of low‐value diagnostic tests [8]. Use of low‐value tests in EDs have been found to result from a combination of cultural influences, fear of consequences, complex decision making, resource constraints, limited clinician abilities and need for efficiency [8]. Patient expectations are frequently cited by emergency clinicians as a cultural driver of low‐value tests [8, 9]. Prior literature has explored expectations in ED patients with specific conditions, such as syncope and first trimester bleed [10, 11]. Diagnostic clarity, empathy and shared decision making were the expectations in patients with syncope [10] whereas patients with first trimester bleed wanted to know whether they were having a miscarriage [11]. Findings from these studies can inform clinician‐patient conversations about diagnostic tests in ED patients with syncope and suspected miscarriage, however, may not be generalisable to other patient cohorts. Expectations of diagnostic tests in ED patients with other illnesses are unknown and therefore need further elucidation.

Fear of adverse consequences has been reported by emergency clinicians [8] to influence the performance of tests to mitigate medicolegal risk, i.e. defensive medicine [12]. In contrast, defensive medicine was viewed as a barrier to therapeutic relationship in a qualitative study of patients with chronic illness [13]. These conflicting views suggest that emergency clinicians' perceptions about patient expectations may differ from reality. As the study by Ries et al. [13] was limited to patients with long‐term illness, an exploration of views of ED patients with acute illness is required to inform emergency medicine practitioners about defensive medicine.

A better understanding of overall patient expectations of ED diagnostic tests could therefore enhance patient engagement, clarify clinician misperceptions, promote clinician‐patient partnership and nurture a culture of diagnostic stewardship [14]. The aim of this study was to explore patient expectations about diagnostic tests to inform diagnostic stewardship in emergency medicine practice.

## 2 Methods

### 2.1 Design

A qualitative, descriptive, study design was chosen to gain an in‐depth understanding of meaning and context of participants' experiences and perspectives [15]. Focus groups were employed to enable exploration of a series of open‐ended questions and encourage participants to explore issues of importance to them by generating their own questions and pursuing their own priorities [16].

### 2.2 Setting

The study was conducted at Townsville University Hospital ED, a major‐referral, mixed ED with an annual patient census of 97,259 in the 2024‐2025 financial year. Townsville is a regional city in Queensland, Australia with a resident population of 234,283 including 9% who identify as Aboriginal and/or Torres Strait Islanders [17].

### 2.3 Participant Selection

Townsville community members aged ≥ 18 years who had been patients at Townsville University Hospital ED within the preceding 24 months were eligible. The study was advertised to eligible participants through e‐mail (hospital consumer advisory council e‐mail groups, Indigenous health liaison officers), social media (hospital Facebook) and posters (ED waiting room, Townsville Aboriginal and Islander Health Service). Recruitment strategies were developed in consultation with community collaborators and hospital consumer engagement team. Interested participants were e‐mailed the participant information and consent sheet with an invitation to ask further questions. A convenience sample of consenting participants was recruited. The final number of focus groups was determined by data adequacy, i.e. sufficient information to comprehensively map variations in patient expectations about diagnostic tests [18].

### 2.4 Data Collection

A focus group guide (Appendix 1, Supplemental file) was employed to ensure a comprehensive coverage of issues with appropriate prompts and probes [19]. The questions in the guide were informed by published literature [8, 12, 20]. The guide was piloted with two community collaborators to refine the structure, content and questioning approach [21]. The focus groups were conducted in‐person in a secure, quiet meeting room at participants' convenience and were facilitated by the study team(one emergency physician, two academic qualitative researchers, two patient representatives). The focus group discussions were recorded using a digital voice recorder.

### 2.5 Data Analysis

The focus group discussions were transcribed by the lead author with assistance of an Artificial Intelligence transcription software (otter ai). The transcribed data was imported into NVivo and subjected to line‐by‐line and axial coding by the lead author. The coded data was subjected to inductive content analysis to generate code categories, subthemes and themes guided by Hsieh & Shannon [19, 22, 23].

### 2.6 Trustworthiness

Reflexivity, audit trail, thick description, and participant validation were the strategies employed to ensure rigour and trustworthiness [19]. Reflexivity involved the lead author being aware of his biases and assumptions due to his gender (male), occupation (emergency physician), experience (9 years post‐fellowship, previously published qualitative research) and interest (study part of a Doctorate in Philosophy exploring the de‐implementation of low‐value diagnostic tests in emergency medicine practice). Audit trail entailed the lead author maintaining a detailed record of transformation of data into codes, categories and themes. The lead author regularly discussed emerging codes and themes with the co‐authors to minimise bias. These discussions occurred through in‐person meetings at 4–6‐week intervals during data analysis. Thick description ensured accurate interpretation and analysis of underlying context and meaning of participant quotes. Participant validation verified that the findings accurately reflected participant views. Feedback was sought on the final manuscript which was shared with participants by e‐mail. Validation resulted in no changes to the manuscript.

### Ethics and Reporting

Townsville Hospital and Health Service Human Research Ethics Committee (AM/2024/QTHS/89509) approved the study. The study report adheres to Consolidated criteria for Reporting Qualitative research guidelines (Appendix 2, Supplemental file).

## 3 Results

Five focus groups, with 22 participants, were conducted between November 2024 and August 2025. Each focus group had 3‐6 participants of mixed gender and age and lasted 60‐90 min. All First Nations participants were in one group. Table 1 details participant characteristics.

**Table 1:** Characteristics of focus group participants in a qualitative study of expectations of diagnostic tests in emergency medicine practice.

Characteristics		Number (proportion^a^) of participants
Age	≤ 30 y	1(5%)
	31–55 y	11(50%)
	56–75 y	10(45%)
	≥ 76 y	0
Gender	Male	6(27%)
	Female	16(73%)
English first language	Yes	21(95%)
	No	1(5%)
Identify as First Nations	Yes	6(27%)
	No	16(73%)
School‐aged children in family unit	Yes	6(27%)
	No	16(73%)
Most recent ED visit	Within 6 months	14(64%)
	Within 12 months	3(14%)
	Within 18 months	1(5%)
	Within 24 months	4(18%)
Primary reason for ED presentation	Head trauma, extremity trauma, cardiac dysrhythmia, presyncope, pneumonia, gastrointestinal bleed, rhabdomyolysis, worsening osteoarthritic pain, elderly fall, ruptured ovarian cyst, appendicitis, perforated diverticulitis, abdominal haematoma, urosepsis, ocular foreign body, extremity foreign body.	22^b^(100%)

^a^ Proportions have been rounded up (if ≥ 0.5) or down (< 0.5) to the nearest integer.

^b^ Some participants had similar reasons for ED presentation.

Participants’ views centred around seeking ED care and diagnostic test expectations.

### 3.1 Seeking ED Care

Individual and systemic factors influenced participants’ decision to attend ED. Concern about worsening illness and inability to find an alternative, affordable, timely care provider prompted ED attendance. Anticipated long wait times, perceived systemic burden by seeking care and limited after‐hours transport led to ED avoidance. Participants exhausted alternative options, including telehealth and urgent care clinics, before ED presentation. Lack of imaging or pathology services at ED substitutive care providers were cited as a driver of ED attendance. Participants appreciated quality of ED care amidst resource constraints. Illustrative participant quotes are presented in Table 2.

**Table 2:** Illustrative focus group participant quotes about ED care in a qualitative study of expectations of diagnostic ******tests in emergency medicine practice.

Themes	Quotes
ED attendance	*“I know the hospital is strained …but it got to the stage where you. physically start falling over and can't walk…end up a burden on the emergency department…’ (P16)*. *‘. we've got us to the non‐emergency care. we got to see a doctor quite quickly…and he said ‘I think you need to go…over to the emergency….(P14.)* *‘…don't go to the urgent clinical care clinic if you need an X ray, because there's no medical imaging on site, they'll send you in your own car to the hospital.’(P2.)* *‘but on the other hand, I'm sitting there thinking, Do you know how hard it is to get into a GP,. how expensive it is.’ (P16)*.
ED avoidance	*‘. there may be other people who don't present because they think they're going to waste time and resource.’ (P3)*. *‘.I've cut myself recently…clearly needed stitches, but there was no way I was going to sit up at that hospital for hours. so I just didn't just bother presenting.’ (P3)*. *‘After hours. is a big issue to get our people home, non‐Indigenous and Indigenous.(P19) they got no phone. no money…(P20) thats why(they) don't want to come up here. There's no way getting home,(P19) so it interferes with their health and well‐being as well to seek help. And then things progress. get worse.(P17)They leave it till it's the worst case scenario before they come in.(P18)*
Appreciation	*‘…And they do listen…they do take time out. I'm really happy.’ (P1)*. *‘I got in not long after. As soon as the nurse came in, she said, You need more pain relief, like was very well done… I was. very impressed.’(P13)*. *‘.I felt like the doctor that was seeing me… I never saw her. supervisor. but I know that she was touching base. she was really thorough…I felt very comforted that I had nothing to worry. even though my heart was doing some strange stuff…(P16)* *‘They work well under pressure…always under the pump… just do a fantastic job, because obviously they've got staff constraints. (P3)*

Abbreviation: P, participant.

### 3.2 Expectations of Diagnostic Tests

Communication, therapeutic relationship, quality of care and patient‐centred care were the key themes of patient expectations of diagnostic tests. These themes are detailed below.

### 3.3 Communication

Participants expected effective communication about rationale and risks‐benefits of tests. Communication was felt to be effective when clinicians listened carefully and provided explanations adapted to patients’ illness severity, health literacy, cognitive abilities and prior experience. Effective communication allayed participant anxiety and increased confidence in clinicians. Knowledge of test rationale, location, implications and confidentiality was thought to be essential for informed choice. Test were considered to be justified when performed to prevent missed diagnoses and provide reassurance. Unjustified tests were perceived to adversely impact patients by prolonging ED stay. Defensive tests were unacceptable as they did not benefit patients. Questioning clinicians about the need for tests came naturally to some participants whilst others found it daunting due to the inherent conflict in challenging the authority of medical experts. Age, life experience and health literacy were felt to influence ability to question clinicians about tests with older patients asking fewer questions. Opinions about the need for risk‐benefit conversation varied based on the perceived risk of a test and prior experience with tests.

### 3.4 Therapeutic Relationship

Empathy, trust and respect were regarded as the foundational pillars of good rapport. Empathetic care encompassed taking concerns seriously, listening to problems and explaining anticipated ED care. Active listening was thought to enable clinicians build a strong rapport. Empathetic care was anticipated to commence from ED triage as a negative experience at triage could lead to distress and disengagement. Participants trusted clinicians to ensure appropriate care and felt disrespected when their concerns were dismissed.

### 3.5 Quality of Care

Timeliness, resources, equity, safety and complexity were the perceived elements of care quality. Participants acknowledged that timely care was influenced by patient (volume, acuity) and systemic (limited staff, space, time) factors. Performing tests to exclude diagnoses was considered reasonable when done equitably. Emergency care deficits were felt to be more pronounced for First Nations patients. Early, appropriate tests before medical assessment were thought to enhance patient safety. Complexity of ED care was thought to influence clinician fallibility.

### 3.6 Patient‐Centred Care

Advocacy, assessment, engagement, experience and vulnerability encompassed patient‐centred care. Participants felt the need for confident communication for self‐advocacy about tests. Participants asserted that tests needed to be tailored to patient presentations, values and preferences. Early tests before medical assessment were supported when targeted to patient symptoms. Communication about tests needed to be adapted to individual cognitive abilities and health literacy. Participants affirmed that patients had the final decision about tests. Participants reported negative experiences when the right tests were not performed at the right time. Staff advocacy was regarded as critical for vulnerable patients to navigate the clinician‐patient power imbalance. Cultural safety was paramount for First Nations participants.

Illustrative participant quotes are presented in Table 3. The coding tree is presented in the supplemental file (Appendix 3).

**Table 3:** Illustrative focus group participant quotes for themes and subthemes of expectations of diagnostic tests in emergency medicine practice.

Themes	Subthemes		Participant quotes
Communication	Effective communication		‘I had bleeding in the stomach…. So they did blood test…urine test, and then…referred me to specialist, and then I had endoscopy done….I find that really excellent…. that somebody actually listened to me.’(P6) ‘Information why they're doing it. We're on this journey together. Tell us why.’ (P11) ‘… And I'm really worried about what's happened here… it's good…to know …what the possibilities might be because… you feel comfortable and confident about what's being done.’ (P3)
	Justification for tests		‘… I'm used to blood tests and X rays…, but knowing why you need it, what they're looking for, if you can tell me in plain English why, the reasons why and the thought process behind it, yes, I'll 100% support you.’(P5) ‘… I think it's a great thing to be doing… extra tests… because amount of times that things…will get missed. when it could have been done with a simple test, you know, just doing an extra ultrasound…’ (P8) ‘And after they did some tests and found that there was nothing dramatic going on, because I was concerned that there was something else going on,’ (P10)
		Defensive testing	‘Oh, test being done to avoid being sued. It never occurred to me, until right now that that happened.’(P7) ‘so they won't be sued? I think that's, I don't think that's the right reason’ (P12) ‘if there's an open conversation happening about the tests that have been done, what the doctor is considering is the issue, and it's all been documented like I think as a patient, if there was something missed, I would still feel confident in the care that I had received.’ (P16)
	Questioning about tests		‘it depends on the attitude and the approach of the doctor too, and the way they're treating you. If you feel you're comfortable and safe to actually ask that question.’(P21) ‘It probably depends a lot on generation and experience. my mum… If she's told that a test is important…She won't question. My younger brother is a lawyer. He would…question everything. My son…he self‐catheterises everyday. His approach is, ‘I'm not an expert. These guys are’. And he wouldn't question at all’ (P2)
	Risk‐benefits		‘I think you do have to give the risk and benefits. It just depends how deeply that person wants to know it.’ (P15) ‘…for…a blood test…,I don't really need somebody to tell me what the risks are…, a urine test, I don't feel like somebody really needs to take the time to explain that… an x ray or a CT scan…it makes sense to understand…the risks.’ (P16) ‘I prefer to have the test done…for example, a cannula or an x ray…. whatever it is, I'd rather find out…. the little bit of pain, inconvenience, discomfort, embarrassment is going to be resolved in the long run…. Urine catheters, on the other hand, scare me. I get very anxious if they say they're going to do a catheter. I've had extreme pain on the two occasions’ (P9)
Therapeutic relationship	Empathy		‘. I think how my daughter and I were treated were 100% better than a lot of other hospitals with how they were treated and dismissed with their concerns of their child. I thought we were really looked after in comparison. But I guess it depends on the human being standing in front of you at that time…with personable skills, bit of empathy goes a long way it does.’ (P11) ‘I was sort of feeling pretty crappy. I said. ‘I think I'm going to pass out’. And then she said, ‘Yeah, no worries’…. it's like she didn't even care.’ (P1)
	Trust		‘community members…would have trust coming here that at least they're going to be supported, or if stuff can't be done here that needs to be included in the discharge summary so their GP can follow up on it or so that the patient knows…’(P20) ‘I have always wanted simple information not hard out literature…this is the contrast … I understand. I could react… I'm like, yeah, no problem…You'll look after me if something happens.’ (P11)
	Respect		‘And I said, I will stay and get the operation done but during that period that I was here, I was treated well, with respect’ (P17) ‘Triage nurse was pretty rude…and it's quite disheartening and unfortunate, because that's the first point of ED and if you get a bad taste in your mouth from that point, you don't want to stay, you don't want to get treated. You want to go.’ (P20) ‘Because it just feels like I'm worthless. It feels like I'm not respected. It feels like my health is not important, because they dismiss my health concerns.’ (P9)
	Rapport		‘… one of the key things was building rapport…a lot of people that come through will always say, no one's listening to me. You really need to listen…just letting the person talk …sometimes…prompt them by asking certain things.’ (P22) ‘… if you take that extra two 5 min time to make rapport with the First Nations consumer, they're going to open up and tell you so much more collateral in the truth and tell you exactly what's going on.’ (P18)
Quality of care	Timeliness		‘So, at 3am I was still waiting…I felt really bad to complain, but same time I was really worried…, so I went up to the counter and I said, Well, what are the chances that you'll be able to get this thing out of the eye?’ (P3) ‘I think if you can say, ‘once we do your bloods…. it's probably going to be about three or 4 h before we even have any answers…When the results come back, we'll talk to you’,…that might maybe calm some people down.’ (P19)
	Resources		‘…I understand from my end exactly how time constraining…everything is. It's just all about …staffing, time management, education, efficiency, equipment availability.’ (P8) ‘. You run out of space, or you run out of people, you run out of resources, or you run out of time. All those things are finite. So you can't keep filling up a bucket.’ (P2)
	Equity		‘ So regardless of the reason…if the resources, for me,. are there, and that's not stopping someone else getting a critical test… do it.’ (P2) ‘… ED, as a whole, indigenous, non‐Indigenous, there's gaps everywhere…definitely, we have more gaps with our own mob.’ (P20)
	Safety		‘…that man over there with really blue legs and overweight and bit sweaty, he doesn't look quite right, but he's not going to…say anything. But if you did a blood test on him, you'd probably find his…mark is all over the shop… it's actually a safety thing’ (P13)
	Complexity		‘… patients, can be really complicated and multifaceted, and you don't always get the true picture of what's going on… it's a teaching hospital when your junior staff…change over…you're constantly always trying to update new staff.’ (P18) ‘…I don't have any perceptions that the doctors and nurses will know everything. I think it's quite complex, and they have to, you know, rely on each other and on the Allied services and tests and things like that. It's not an easy thing.’ (P7) ‘ … you hope that the doctor's gonna get it right,…and get it right the first time. Yeah, that is something that's on people's minds…(P22)
Patient‐centred care	Advocacy	Self advocacy	‘…I would definitely…advocate for myself. And if they were sending me for a test, I'd be asking why…almost to the point where I'd be saying, you tell me now or I'm not having that test.’ (P7) ‘but if you feel strongly…you still have to have… the communication skills, the confidence… quite…challenging’ (P13)
		External advocacy	‘… we've got family members now who are very vocal in… advocating for the patient, for CT scan…’ (P17) ‘I think every First Nations person needs an advocate… because you still face gaps in the system, judgement…stigma.’ (P18)
	Assessment		‘… if it was explained, I'd have no issue, bloods and urine. But…depending on what the presentation is as well.’ (P18) ‘I think if you…give your symptoms to the triage nurse…say it's abdominal pain, they'll go, ‘Okay, well, we'll do…bloods, urine, X‐ray, so get those done by the time the doctor comes.’ (P14)
	Engagement		‘…but then that's that communication… that you know about in this instance this is the communication I need to have. I can't because of this. I guess each case needs to be looked at on its own.’ (P5) ‘As long as the staff have explained to you why, like, you say you don't want it, and they tell you why you should have it…You're sure that the patient fully understands, then you still have to listen to that person's choice…,’ (P21)
	Experience		‘So, experience has taught me to just advocate for myself and to say, look, this has happened before. This has been going on for a certain amount of time. I need to get an answer. Can I get it here, or do I have to go and get it privately?’ (P7) ‘…I wasn't a frequent flyer, I wasn't drug seeking…you could actually see from photos of my abdomen how much it had swollen, etc, and, like, where is the accountability? Why weren't tests done?’ (P9)
	Vulnerability	Cultural safety	‘I do know with family members, they are quite scared of speaking up because they're worried about judgement and stigma and racism’ (P18) ‘And then thank God, the ILO(indigenous liaison officer) came, and even though I felt a bit uncomfortable, well, she was only person I felt safe with.’(p19)
		Power imbalance	‘… I requested… x ray on the back of my knee…when I came with the ambulance, and basically got doctors telling you, ‘You don't need it’. So, what is it? Oh, you know. And then just take their word, because they're the doctors.’ (P19) ‘haven't really had that circumstance, because when the doctor proposes the testing, I'm always quite for it, saying, well, if, if you think that that's what we need to do, then, yeah, let's have a look at that’ (P14)
		Health literacy	‘Some patients are really good historians…They know the medications they're on. They know exactly what's happened to them… And then others are terrible…they can't even name one of the meds they're on. So, I feel like some of the tests that they do are trying to figure out what the patient's probably actually there for’ (P5) ‘A lot of people come here and not really interested at all. They don't really want to know too much about exactly what's going…’ (P4)

Abbreviation: P, participant.

## 4 Discussion

Patients have a spectrum of expectations of ED diagnostic tests based on individual life experiences. Patient expectations are intricately linked to their ED care needs. The following discussion is therefore anchored to patients’ ED care journey.

### 4.1 Before Assessment

ED triage was viewed as a critical juncture for clinicians (medical and nursing) to lay the groundwork for a collaborative partnership built on respectful, empathetic, culturally safe, communication that fosters trust and rapport. Consistent with prior evidence [24], prolonged wait was an issue for some participants whose vulnerability was compounded by anxiety, uncertainty, untreated pain, hunger and other agitated patients in ED waiting room. Open communication about reasons for delay ‐ access block, patient volume, patient surge ‐ was suggested to manage expectations. Symptom‐targeted pathology and imaging tests performed whilst awaiting medical assessment were regarded as efficient. Furthermore, diagnostic tests which align with patient symptoms and likely differential diagnoses could contribute to optimal use and stewardship of tests.

### 4.2 During Assessment

Empathetic listening was considered essential for clinicians to incorporate patient values and preferences into decision‐making about tests that matched symptoms and addressed concerns. Explanation of rationale for tests was felt to be necessary, however, this was not a consistent patient expectation. This need for communication was mirrored in a Canadian qualitative study where hospitalised patients expected information about the rationale and frequency of blood tests [25]. An Australian qualitative study of emergency clinicians and patients reinforced this expectation for communication, however, found that patients received inconsistent and variable communication about diagnostic tests from clinicians [26]. Dahm et al. emphasise that effective communication is influenced by systemic (time, staffing) and individual (clinicians’ stage of clinical reasoning; patients’ perceived anxiety/interest/literacy) factors. Strategic navigation of such contextual challenges is essential to ensure patient‐centred diagnostic tests. The trust and rapport fostered by effective communication could enable clinicians to gather higher quality illness‐related data from patients and minimise the likelihood of diagnostic error.

Divergent views emerged about discussion of risks and benefits of diagnostic tests. Whilst some participants preferred to know, others trusted doctors to choose the right tests. Pain during blood tests was a concern for participants, a finding noted by other investigators [25]. However, some felt that discomfort of needles was a small price to pay compared to the reassurance provided by blood tests. This preference for tests despite associated risk of harm has been described by Swarup et al. [27] In this qualitative study of ED patient expectations of pulmonary embolism testing, patients preferred imaging over clinical examination [27]. Risks were experienced on a spectrum in our study with CT scans being perceived to be higher risk than blood and urine tests. Differential patient views about risks/benefits have also been replicated in cross‐sectional studies of ED patient preferences for tests in hypothetical scenarios of chest pain and mild traumatic brain injury [28, 29]. Conversations about diagnostic tests will therefore need to be tailored to individual patients and tests. Individualised care was indeed a key patient expectation in the study by Swarup et al. [27]

Tests performed to reduce the risk of medicolegal liability, i.e. defensive testing [30], surprised some participants whilst others acknowledged them as a systemic issue driven by clinician concern about litigation. Defensive tests were felt to be inconsistent with patient‐centred care. This view has been echoed by Ries et al. where patients reported that defensive medicine has a negative impact on clinician‐patient relationship [13]. Defensive tests were viewed by our participants to be distinct from symptom‐based tests performed to avoid missing diagnoses. Some participants expected clinicians to not miss diagnoses whilst recognising that this was unrealistic due to human fallibility and complexity of ED care. Our participants suggested that clinicians could overcome the urge for defensive testing by thorough patient assessment accompanied by transparent communication, including documentation, of clinical reasoning and decision‐making.

The Choosing Wisely Australia initiative encourages patients to ask questions to ensure the right tests [20]. Participants in our study were comfortable asking questions, declining a recommended test and seeking non‐ED providers if they were convinced of the need for tests. Vulnerability was felt to limit participants’ ability to question clinicians. Elderly and Indigenous patients were highlighted as groups that would rarely challenge the need for tests due to faith in the medical profession and power imbalance. Clinician‐patient power imbalance turned disagreements with clinicians into conflict‐prone situations. Conflicts about tests were highlighted as challenging even by our participants who felt confident to stand up for themselves. Advocacy by ED staff, including Indigenous Liaison Officers, was suggested as a strategy to ensure cultural safety and to empower patients to handle conflict. Conflict resolution was thought to require a collaborative approach underpinned by effective communication about rationale for/against tests whilst addressing patient concerns. Riganti et al. concur with this ‘align‐acknowledge‐refocus’ approach of active discouragement when harms outweigh benefits whilst co‐creating solutions when evidence is inconclusive [31].

Although shared decision making was not identified as a theme, elements of shared decision‐making, as described by Schoenfeld et al. (collaborative decision‐making, risk‐benefit communication, consideration of patient values/preferences/experience) [32], were present across the four themes of patient expectations.

### 4.3 After Assessment

Anticipatory anxiety was a key participant concern whilst awaiting test results. Explanation about the estimated wait time for results and anticipated course of clinical care was thought to improve patient outcomes by allaying anxiety, enabling alternative plans and maintaining engagement. Participants acknowledged that some tests were non‐emergent and expected continuity of care through a clear primary care follow‐up plan. This need for clarity in ED discharge communication has been highlighted in prior literature [33]. Clinician‐patient discussions about the timing of tests (emergent vs non‐emergent) can assist the choice of right tests for the right patients at the right time thus promoting resource stewardship.

### 4.4 Communication Is Crucial

Communication is the common thread that runs through our participant expectations of diagnostic tests. Poor clinician‐patient communication and absence of person‐centred care drive low‐value care [34]. Individualised communication is essential as identical messages can have starkly different impact depending on patient mindsets [35]. Test‐related communication will need to be tailored to patient values, views and beliefs that influence expectations [36]. Effective communication can enable emergency clinicians gather higher quality data to inform pre‐test probability and patient‐centred decision‐making about tests. A nuanced clinical approach will be necessary to meet the competing needs of effective communication and efficient emergency care [37].

### 4.5 Strengths and Limitations

The inclusion of First Nations and regional community members as participants are strengths as this provided important cultural perspectives and strengthened the breadth of views captured. However, our focus groups did not include participants from other culturally and linguistically diverse communities, a potentially vulnerable group who may experience cultural and language barriers that limit communication of expectations.

Age‐group imbalance (one under 30 y, none over 75 y) is mitigated by our participants’ views as caregivers for their children and parents. The biases due to single‐centre design, gender imbalance (mostly female), high health literacy and power imbalance are minimised by the frank expression of broad range of positive and negative experiences of ED care.

### 4.6 Implications

The findings of our study indicate that personalised communication, rather than a one‐size‐fits‐all approach, will be necessary to ensure that diagnostic tests address ED patient symptoms and concerns. Effective, patient‐centred, communication ought to be a core tenet of future endeavours to promote value‐based diagnostic tests in emergency medicine practice. Future research could explore effective strategies to nurture patient‐centred, culturally safe, test communication skills amongst emergency medicine practitioners.

## 5 Conclusion

Respectful, trusting, empathetic, culturally safe, communication was the overarching patient expectation about diagnostic tests. Effective communication can facilitate a clinician‐patient partnership which empowers patients to make an informed choice about diagnostic tests. This partnership is critical in guiding emergency clinicians and patients towards a culture of diagnostic stewardship.

## Author Contributions

**Vinay Gangathimmaiah:** writing – original draft, conceptualisation, methodology, software, data curation, investigation, formal analysis. **Rebecca Evans:** writing – review and editing, methodology, supervision, validation. **Tarun Sen Gupta:** writing – review and editing, supervision. **Karen Cupitt:** writing – review and editing, methodology, validation. **Sharon Oakley:** writing – review and editing. **Karen Carlisle:** writing – review and editing, methodology, supervision, validation.

## Funding

The authors received no specific funding for this work.

## Ethics Statement

Townsville Hospital and Health Service Human Research Ethics Committee (AM/2024/QTHS/89509) approved the study.

## Conflicts of Interest

The authors declare no conflicts of interest.

## Supporting information

DELVE_Phase3a_Supplemental_File_01022026.

## Data Availability

The data that support the findings of this study are available on request from the corresponding author. The data are not publicly available due to privacy or ethical restrictions.

## References

[1] E. W. Verkerk , M. A. C. Tanke , R. B. Kool , S. A. van Dulmen , and G. P. Westert , “Limit, Lean or Listen? A Typology of Low‐Value Care That Gives Direction in De‐Implementation,” International Journal for Quality in Health Care 30, no. 9 (2018): 736–739, 10.1093/intqhc/mzy100. 29741672 PMC6307334

[2] I. A. Scott , A. G. Elshaug , and M. Fox , “Low Value Care Is a Health Hazard That Calls for Patient Empowerment,” Medical Journal of Australia 215, no. 3 (2021): 101, 10.5694/mja2.51168. 34275155

[3] H. Walker , C. West , L. Lawton , T. I. Emeto , and V. Gangathimmaiah , “Could Low‐Value Diagnostic Tests Be Compounding Access Block? A Single‐Site, Cross‐Sectional Study,” Emergency medicine Australasia: EMA 37, no. 4 (2025): e70100, 10.1111/1742-6723.70100. 40686189 PMC12278031

[4] Information CIfH . Overuse of Tests and Treatments in Canada‐ Progress Report. 2022.

[5] S. Judkins , “Emergency Department in Perspective: How Emergency Department Culture Drives Over‐Investigation,” Emergency Medicine Australasia: EMA 37, no. 2 (2025): e70015, 10.1111/1742-6723.70015. 40045776

[6] K. C. Claeys , K. C. Coffey , and D. J. Morgan , “What Is Diagnostic Stewardship?,” The Journal of Applied Laboratory Medicine 10, no. 1 (2025): 130–139, 10.1093/jalm/jfae130. 39749441

[7] D. J. Morgan , P. N. Malani , and D. J. Diekema , “Diagnostic Stewardship to Prevent Diagnostic Error,” Journal of the American Medical Association 329, no. 15 (2023): 1255–1256, 10.1001/jama.2023.1678. 36862424

[8] V. Gangathimmaiah , R. Evans , N. Moodley , T. Sen Gupta , and K. Carlisle , “Drivers of Low‐Value Diagnostic Tests in Emergency Medicine Practice: A Qualitative Descriptive Study,” Emergency Medicine Journal 42, no. 8 (2025): 503–510, 10.1136/emermed-2024-214241. 40037782 PMC12322463

[9] H. K. Kanzaria , J. R. Hoffman , M. A. Probst , D. Harris , S. Berry , and R. H. Brook , “Emergency Physician Perceptions of Unnecessary Advanced Diagnostic Imaging: A National Survey Study. Conference Abstract,” Academic Emergency Medicine 21, no. 5 (2014): S227–S228, 10.1111/acem.12365. 25807868

[10] J. Strommen , L. Masullo , T. Crowell , and P. Moffett , “First‐Trimester Vaginal Bleeding: Patient Expectations When Presenting to the Emergency Department,” Military Medicine 182, no. 11 (2017): e1824–e1826, 10.7205/milmed-d-17-00108. 29087848

[11] J. M. Clouser , M. Sirrine , C. A. McMullen , et al., “Passing Out Is a Serious Thing”: Patient Expectations for Syncope Evaluation and Management,” Patient Preference and Adherence 15 (2021): 1213–1223, 10.2147/ppa.S307186. 34113084 PMC8187096

[12] N. M. Ries , B. Johnston , and J. Jansen , “A Qualitative Interview Study of Australian Physicians on Defensive Practice and Low Value Care: “It's Easier to Talk about Our Fear of Lawyers Than to Talk about Our Fear of Looking Bad in Front of Each Other,” BMC Medical Ethics 23, no. 1 (2022): 16, 10.1186/s12910-022-00755-2. 35246129 PMC8895622

[13] N. M. Ries , B. Johnston , and J. Jansen , “Views of Healthcare Consumer Representatives on Defensive Practice: ‘We Are Your Biggest Advocate and Supporter Not the Enemy,” Health Expectations 25, no. 1 (2022): 374–383, 10.1111/hex.13395. 34859547 PMC8849368

[14] E. E. Sypes , C. De Grood , F. M. Clement , et al., “Understanding the Public's Role in Reducing Low‐Value Care: A Scoping Review,” Implementation Science 15, no. 1 (2020): 20, 10.1186/s13012-020-00986-0. 32264926 PMC7137456

[15] J. W. Creswell and C. N. Poth , Qualitative Inquiry and Research Design Choosing Among Five Approaches (Sage, 2024). *5th ed* .

[16] J. Kitzinger , “Qualitative Research. Introducing Focus Groups,” BMJ (London) 311, no. 7000 (1995): 299–302, 10.1136/bmj.311.7000.299. PMC2550365 7633241

[17] Statistics ABo . Townsville Census All Persons QuickStats, accessed 11 October, 2025, https://www.abs.gov.au/census/find-census-data/quickstats/2021/318.

[18] H. M. Levitt , S. L. Motulsky , F. J. Wertz , S. L. Morrow , and J. G. Ponterotto , “Recommendations for Designing and Reviewing Qualitative Research in Psychology: Promoting Methodological Integrity,” Qualitative Psychology 4, no. 1 (2017): 2–22, 10.1037/qup0000082.

[19] P. Liamputtong , Qualitative research methods (Oxford University Press, 2012). 5th ed.

[20] Australia. CW . 5 Questions to Ask Your Doctor or Healthcare Provider Before You Get any Test, Treament or Procedure, accessed 11 October, 2025, https://www.choosingwisely.org.au/resources/consumers-and-carers/5questions.

[21] R. L. Breen , “A Practical Guide to Focus‐Group Research,” Journal of Geography in Higher Education 30, no. 3 (2006/11/01 2006): 463–475, 10.1080/03098260600927575.

[22] H. F. Hsieh and S. E. Shannon , “Three Approaches to Qualitative Content Analysis,” Qualitative Health Research 15, no. 9 (2005): 1277–1288, 10.1177/1049732305276687. 16204405

[23] D. F. Vears and L. Gillam , “Inductive Content Analysis: A Guide for Beginning Qualitative Researchers,” Focus on Health Professional Education: A Multi‐Professional Journal 23, no. 1 (2022): 111–127, 10.11157/fohpe.v23i1.544.

[24] D. Kuhn , P. S. Pang , B. R. Hunter , et al., “Patient Comments and Patient Experience Ratings Are Strongly Correlated With Emergency Department Wait Times,” Quality Management in Health Care 33, no. 3 (2024): 192–199, 10.1097/qmh.0000000000000460. 38941584

[25] S. Pokharel , Z. Khawaja , J. Williams , et al., “Patient Perceptions of In‐Hospital Laboratory Blood Testing: A Patient‐Oriented and Patient Co‐Designed Qualitative Study,” Health Expectations: An International Journal of Public Participation in Health Care and Health Policy 27, no. 1 (2024): e13880, 10.1111/hex.13880. 37751312 PMC10726148

[26] M. R. Dahm , J. Li , J. Thomas , P. Smith , and A. Georgiou , “How Is Test‐Related Information Communicated in Australian Emergency Departments? ‐ Ed Clinicians’ and Patients’ Perspectives,” Patient Education and Counseling 104, no. 8 (2021): 1970–1977, 10.1016/j.pec.2021.01.009. 33500178

[27] V. Swarup , A. Soomro , S. Abdulla , and K. de Wit , “Patient Values and Preferences in Pulmonary Embolism Testing in the Emergency Department,” Academic Emergency Medicine 29, no. 3 (2022): 278–285, 10.1111/acem.14400. 34661318

[28] J. D. Porath , A. P. Meka , C. Morrow , et al., “Patient Preferences for Diagnostic Testing in the Emergency Department: A Cross‐Sectional Study,” Academic Emergency Medicine 25, no. 6 (2018): 627–633, 10.1111/acem.13404. 29505177 PMC5995656

[29] J. Padalecki , K. T. Xu , C. Smith , L. Carrasco , J. Hensley , and P. B. Richman , “How Much Risk Are Emergency Department Patients Willing to Accept to Avoid Diagnostic Testing,” Turkish Journal of Emergency Medicine 17, no. 1 (2017): 16–21, 10.1016/j.tjem.2016.09.009. 28345068 PMC5357107

[30] D. M. Studdert , “Defensive Medicine Among High‐Risk Specialist Physicians in a Volatile Malpractice Environment,” Journal of the American Medical Association 293, no. 21 (2005): 2609–2617, 10.1001/jama.293.21.2609. 15928282

[31] P. Riganti , K. S. Kopitowski , K. McCaffery , and L. van Bodegom‐Vos , “The Paradox of Using Sdm for De‐Implementation of Low‐Value Care in the Clinical Encounter,” BMJ Evid Based Med 29, no. 1 (2024): 14–16, 10.1136/bmjebm-2022-112201. 37080738

[32] E. M. Schoenfeld , H. K. Kanzaria , D. D. Quigley , et al., “Patient Preferences Regarding Shared Decision Making in the Emergency Department: Findings From a Multisite Survey,” Academic Emergency Medicine 25, no. 10 (2018): 1118–1128, 10.1111/acem.13499. 29897639 PMC6185792

[33] T. L. Alberti and A. Nannini , “Patient Comprehension of Discharge Instructions From the Emergency Department: A Literature Review,” Journal of the American Association of Nurse Practitioners 25, no. 4 (2013): 186–194, 10.1111/j.1745-7599.2012.00767.x. 24218236

[34] E. W. Verkerk , J. A. H. Boekkooi , E. G. M. Pels , and R. B. Kool , “Exploring Patients’ Perceptions of Low‐Value Care: An Interview Study,” Patient Education and Counseling 111 (2023): 107687, 10.1016/j.pec.2023.107687. 36958071

[35] G. Gabay , A. Gere , G. Zemel , and H. Moskowitz , “Personalized Communication With Patients at the Emergency Department‐An Experimental Design Study,” Journal of Personalized Medicine 12, no. 10 (2022): 1542, 10.3390/jpm12101542. 36294684 PMC9605307

[36] T. Rozbroj , R. Haas , D. O'connor , et al., “How Do People Understand Overtesting and Overdiagnosis? Systematic Review and Meta‐Synthesis of Qualitative Research,” Social Science & Medicine (1982) 285 (2021): 114255, 10.1016/j.socscimed.2021.114255. 34391966

[37] M. Miao , M. R. Dahm , J. Li , J. Thomas , and A. Georgiou , “Managing Uncertainty During the Communication of Diagnostic Test Information Between Patients and Clinicians in Australian Emergency Care,” Qualitative Health Research 30, no. 8 (2020): 1287–1300, 10.1177/1049732320913037. 32249721

