# Supplementary material for: ‘Communication Is Crucial’: A Qualitative Study of Patient Expectations of Diagnostic Tests in Emergency Medicine Practice
Source: Health Expect. 2026 Mar 26;29(2):e70648. doi: 10.1111/hex.70648 (PMC13045229; doi:10.1111/hex.70648)
Supplement: Supplementary file 1 — DELVE_Phase3a_Supplemental_File_01022026. [file HEX-29-e70648-s001.docx]

**Appendix 1. Focus group guide for a qualitative study of patient expectations of diagnostic tests in emergency medicine practice.**

**Preamble**

Greeting: Hi everyone, Thank you for making the time to be part of the focus group today. We really appreciate your time.

Purpose: First, a little bit about why we are doing this focus group. We want to understand your thoughts and experiences about care that you have received or expect to receive when you turn up to Townsville Emergency Department. The information collected in this focus group will be used to inform Townsville emergency doctors and nurses about how they can provide better care. As these focus groups are part of a research project, the de-identified information will also be shared widely through different scientific and community forums to enable conversations between community members and emergency department staff across Queensland and Australia about better emergency care.

Content and structure: We expect the focus group to last 45 - 60 minutes. We will be listening, making notes and recording our conversation on digital voice recorders.

Ethics: Everything you say today is confidential within the group and will only be used for the purpose of this. To ensure confidentiality, we request you not to share today’s discussion outside the group. All identifying details will be removed before using the information. If you feel like taking a break at any time, please let us know and we will pause the focus group. If you do not feel comfortable answering a question, please let us know and we will move on to the next question. If you would like to stop being part of the focus group and withdraw consent, please let us know.

Clarify understanding and confirm consent: Do you have any questions about what has been said so far? Can you please confirm that I have your consent for the focus group?

Introduce group members: Could we go around the group and introduce ourselves?

Before we begin the interview, would you like to tell us or ask us anything? Let us begin. (Commence recording.)

**Main body**

1. The following questions are about your expectation when you present to our Emergency Department (ED). How do you feel about
   - 1. ED doctors having answers to your questions every time?
     2. ED doctors to be right every time and giving definitive and clear answers?
     3. ED doctors never missing a condition?

Prompt: Do you have any feedback about the above questions?

1. Our emergency department doctors sometimes order tests to find out what is going on. The following questions are about your thoughts about such tests.
   1. What are your expectations about tests being ordered every time you present to our emergency department?
      1. If not, when you do expect tests?
   2. What do you prefer to know before undergoing tests in our Emergency Department?
   3. What do you think about tests being ordered for the following reasons?
      1. Doctors' concern about receiving a complaint/legal action/reputation damage.
      2. Doctors' concern about being reported to/investigated by the Australian medical board or health ombudsman.
      3. Doctors not having enough physical space to talk to and examine patients.
      4. Doctors not having enough time to talk to and examine patients.
      5. Doctors being tired/ busy.
   4. What do you think about tests being done in situations where harms of tests outweigh benefits? Harms may include pain due to unnecessary needles from blood tests, cancer risk due to unnecessary radiation, long ED wait times and anxiety and distress due to false positive test results.
   5. How do you feel about asking the following questions to doctors before you get a test in our Emergency Department?
      1. Do I really need this test?
      2. What are the risks of having the test?
      3. Are there simpler and safer alternative options to having the test?
      4. Will I get worse or better if I don’t have this test?
      5. What are the emotional, time and financial costs of this test to me?
   6. How do you feel about situations where there is disagreement between yourself and the doctors about whether a test needs to be done or not done?
2. Do you have any other suggestions about how our Emergency Department staff can provide better care to patients?

**Close**

That concludes the focus group. We may need to contact you again to clarify some of the things we have discussed today and confirm that our interpretation of your thoughts are accurate. Would that be alright?

We have no further questions. Is anything else that you would like to bring up that we have not discussed or touched upon?

Thank you again for making the time.

**Appendix 2. COREQ checklist for a qualitative study of patient expectations of diagnostic tests in emergency medicine practice.**

| **Item No** | | **Guide Questions/Description** | **Reported on Page #** |  |
| --- | --- | --- | --- | --- |
| **Domain 1: Research team and reflexivity** | | | |  |
| **Personal Characteristics** | | | |  |
| 1. Interviewer/ facilitator | | Which author/s conducted the interview or focus group? | Pg 6 |  |
| 2. Credentials | | What were the researcher’s credentials? E.g., PhD, MD | Pg 6 |  |
| 3. Occupation | | What was their occupation at the time of the study? | Pg 6 |  |
| 4. Gender | | Was the researcher male or female? | Pg 6 |  |
| 5. Experience and training | | What experience or training did the researcher have? | Pg 6 |  |
| **Relationship with participants** | | | |  |
| 6. Relationship established | | Was a relationship established prior to study commencement? | No |  |
| 7. Participant knowledge of the interviewer | | What did the participants know about the researcher? e.g. personal goals, reasons for doing the research? | Pg 5 |  |
| 8. Interviewer characteristics | | What characteristics were reported about the interviewer/facilitator? e.g. Bias, assumptions, reasons and interests in the research topic | Pg 6 |  |
| **Domain 2: Study design** | | |  |  |
| **Theoretical framework** | | |  |  |
| 9. Methodological orientation and Theory | What methodological orientation was stated to underpin the study? e.g. grounded theory, discourse analysis, ethnography, phenomenology, content analysis | Pg 6 |  |  |
| **Participant selection** | | |  |  |
| 10. Sampling | How were participants selected? e.g., purposive, convenience, consecutive, snowball | Pg 5 |  |  |
| 11. Method of approach | How were participants approached? e.g., face-to-face, telephone, mail, email | Pg 5 |  |  |
| 12. Sample size | How many participants were in the study? | Pg 7 |  |  |
| 13. Non-participation Setting | How many people refused to participate or dropped out? Reasons? | None |  |  |
| 14. Setting of data collection | Where was the data collected? e.g., home, clinic, workplace | Pg 6 |  |  |
| 15. Presence of nonparticipants | Was anyone else present besides the participants and researchers? | No |  |  |
| 16. Description of sample | What are the important characteristics of the sample? e.g. demographic data, date | Pgs 7,19 |  |  |
| **Data collection** | | |  |  |
| 17. Interview guide | Were questions, prompts, and guides provided by the authors? Was it pilot tested? | Pg 6 and Appendix 1, Supplemental file |  |  |
| 18. Repeat interviews | Were repeat interviews carried out? If yes, how many? | No |  |  |
| 19. Audio/visual recording | Did the research use audio or visual recording to collect the data? | Pg 6 |  |  |
| 20. Field notes | Were field notes made during and/or after the interview or focus group? | No |  |  |
| 21. Duration | What was the duration of the interviews or focus group? | Pg 7 |  |  |
| 22. Data saturation | Was data saturation discussed? | Pg 5 |  |  |
| 23. Transcripts returned | Were transcripts returned to participants for comment and/or correction? | No |  |  |
| **Domain 3: Analysis and findings** | | |  |  |
| **Data analysis** | | |  |  |
| 24. Number of data coders | How many data coders coded the data? | Pg 6 |  |  |
| 25. Description of the coding tree | Did the authors provide a description of the coding tree? | Pg 10 and Appendix 2, Supplemental file |  |  |
| 26. Derivation of themes | Were themes identified in advance or derived from the data? | Pg 6 |  |  |
| 27. Software | What software, if applicable, was used to manage the data? | Pg 6 |  |  |
| 28. Participant checking | Did participants provide feedback on the findings? | Pgs 7 |  |  |
| **Reporting** | | |  |  |
| 29. Quotations presented | Were participant quotations presented to illustrate the themes/findings? Was each quotation identified? e.g., participant number | Pgs 20-24 |  |  |
| 30. Data and findings consistent | Was there consistency between the data presented and the findings? | Pgs 8-15 , 20-24 |  |  |
| 31. Clarity of major themes | Were major themes clearly presented in the findings? | Not applicable |  |  |
| 32. Clarity of minor themes | Is there a description of diverse cases or a discussion of minor themes? | Not applicable |  |  |

* Developed from: Tong A, Sainsbury P, Craig J. Consolidated criteria for reporting qualitative research (COREQ): a 32-item checklist for interviews and focus groups. International Journal for Quality in Health Care. 2007. Volume 19, Number 6: pp. 349 – 357

**Appendix 3. Coding tree for themes and subthemes of patient expectations of diagnostic tests in emergency medicine practice.**

| **Themes** | **Subthemes** | **Code Categories** | **Codes** |
| --- | --- | --- | --- |
| Communication | Effective communication | Elements of effective communication | Detailed documentation being part of societal accountability  Explaining rationale of repetitive questioning enabling better therapeutic relationship  Explaining test rationale part of clinician-patient shared care journey  Feeling stressed when ED staff do not listen  Feeling that staffing constraints and case loads impact quality of communication  Interlinked electronic medical records reducing unnecessary tests  Listening to patients  Listening to patients being crucial  Needing to know the rationale of repetitive questions by different ED clinicians  Not having enough information about ED care  Not knowing patient history leading to ED staff setting unrealistic goals  Patients' caregivers being able to provide adequate history  Patients educating staff about their illness  Patient having difficulty understanding explanations about tests |
|  |  | Importance of effective communication | Benefits of good communication about tests worth the short time investment  Communication about care to patients being critically important  Communication about long waiting times being of use  Communication being an important aspect of ED care  Communication being necessary for patient education and health literacy  Communication rather than quality of care being an area of improvement for ED staff  Effective clinician-patient communication resulting in optimal outcomes  Emphasising the importance of communication and holistic care despite the busyness in ED  Emphasising the need for intradepartmental communication to optimise ED care  Listening to patients enabling better patient care  Poor intradepartmental communication leading to patient and family anxiety  Simple and clear communication about triage being important  Transparent communication about tests putting patients at ease  Transparent communication important for patients to understand what is happening with their bodies |
|  |  | Effective communication about care | Accepting delay in ED care with good communication about reason for delay  Appreciating being informed about all aspects of care  Appreciating generational shift of medical profession towards better communication  Appreciating ongoing communication about care  Being satisfied with honest communication about care  Communicating with patients in layperson language  Giving patients time enables them to volunteer all relevant information  Wanting to be informed about all aspects of care |
|  |  | Effective communication about tests | Being informed about tests  Being informed about tests in layperson language  Being satisfied about doctors' explanation about tests  Communicating confidentiality of test results to patients  Communicating plan for non-urgent tests  Communicating the significant time delay and implication of test results  Communication about tests essential for patients who might not want tests for non-medical reasons  Communication overcoming patient anxiety due to tests  Communication of test rationale helping patients' anxiety and uncertainty  Communication of test results being an important part of care  Confirming comprehension about tests being an important aspect of communication  Contrasting the information provision about tests at two facilities  Emphasising the significance communication about the rationale and implications of tests  Explicitly discussing whether tests make a difference along with pros and cons  Wanting ED staff to listen to concerns prior to doing tests  Wanting to know the results and implications of the tests with context  Wanting to know the wait time for and location of tests prior to having tests |
|  |  | Ineffective communication about care | Absence of communication to First Nations family about critical patient status  Being disappointed about the lack of communication about interfacility transfers  Being disturbed by poor intradepartmental communication which triggered patient and family anxiety  Being scared by adverse event due to poor intradepartmental communication  Communication being ineffective with the use of medical jargon  ED staff being unaware of patients due to poor interfacility communication  ED staff not communicating about care  ED staff not listening to patients  Inadequate interfacility communication adversely impacting patient care  Poor communication resulting in adverse patient outcome  Suboptimal care resulting from poor communication at care handovers |
|  |  | Ineffective communication about tests | Accepting miscommunication about tests and results from ED staff without complaining  Acknowledging that tests happen without communication  Feeling poor communication about tests is due to lack of staff time  Patients getting anxious following communication about tests  Test results being not relayed to patients |
|  | Justification |  | Acknowledging that unnecessary tests are happening  Appreciating detailed information about rationale and implications of tests  Being swayed towards testing for family due to guilt  Being swayed towards testing for family due to medical recommendation  Being upset for doing tests that are not clinically indicated  Being swayed towards testing due to organisational policy  Cost of tests justified if reassuring patients  Easy availability of tests leading to unjustified tests  ED tests due to insufficient space or staff or time being a challenge due to finite resources  ED tests due to insufficient space  ED tests due to insufficient staff  ED tests due to insufficient time  Feeling tests should be performed based on symptoms  Feeling unnecessary tests should not be done  Negative tests being of help as much as positive tests  Not wanting to know the rationale of tests prior to tests  Nurses explaining rationale of tests done prior to medical assessment  Questioning the value of diagnostic tests in palliative patients  Rational testing in complex patients being a challenge due to diagnostic uncertainty  Refusing a test without reasonable justification by ED staff  Supporting tests that are justified in layperson language  Tests based on patient symptoms being optimal ED care  Tests being done to determine reason for ED visit if insufficient history  Tests preventing missed diagnoses  Tests reassuring patients  Tests to rule out diagnoses being justified  Unjustified tests impacting timely ED care for other patients  Unjustified tests prolonging patient ED stay  Wanting to know the implications of a diagnostic test  Wanting to know the need for additional tests in ED  Wanting to know the rationale of tests even if complexities involved  Wanting to know the results of tests regardless of the implications |
|  |  | Defensive  testing | Acknowledging that a minority of patients may be litigious  Acknowledging that concern about litigation drives diagnostic tests  Acknowledging that defensive tests are a reality  Acknowledging the prevalence of litigiousness in the community  Avoiding defensive testing by clinical decision-making and justification of test performance  Avoiding defensive testing by documentation of open conversations about the rationale for/against tests  Avoiding defensive testing by thorough understanding of patients' past history  Avoiding defensive testing by reasonable conversations about rationale for/against tests  Avoiding defensive tests in palliative patients through open communication  Avoiding defensive tests through communication about test utility  Defensive tests being a cultural and systemic issue  Defensive testing being driven by senior clinicians  Defensive tests being rare  Defensive tests having no value to patients or health system  Defensive tests not being patient centred  Defensive tests prolonging patient ED length of stay  Detailed history being a good defence to justify omission or commission of tests  Diagnostic testing based on best available information to overcome defensive testing  Disagreeing with tests being done as part of defensive medicine  Early open conversations with patients and family critical in avoiding defensive testing  Effective communication about tests being a good defence for missed diagnoses  Emphasising that tests should be done for a legitimate medical reason  Fear of litigation driving tests  Feeling sad about the existence of defensive testing  Feeling strongly against tests for medicolegal reasons  Feeling tests should not be done for medicolegal reasons  Feeling that defensive testing is a challenging issue  Feeling that defensive tests contribute to access block  Feeling that defensive tests might pick up the underlying pathology  Feeling that education may help overcome the urge for defensive testing  Feeling that unresolved past negative experiences may be driving defensive testing  Litigious patients may be driving a culture of defensive testing  Not being aware that tests are done as part of defensive medicine  Not wanting tests driven by fear of litigation  Ordering ED tests based on patient symptoms being the best defence against complaints and lawsuits  Pedantic documentation about decisions to overcome defensive testing  Questioning performance of defensive tests that do not improve patient care  Questioning the non-performance of tests in a culture of defensive testing  Risks of defensive tests outweighing benefits  Strongly agreeing that tests are being done as defensive medicine  Testing based on clinical acumen rather than medicolegal reasons |
|  | Questioning |  | Attitude of doctors influencing patient comfort and safety to question tests  Being aware of the choosing wisely questions about value-based care  Being comfortable asking for a necessary test from a doctor  Being comfortable questioning doctors about tests if insufficient information  Being scared to ask questions of doctors about tests  Comfort in questioning doctors influenced by vulnerability  Knowledge empowering patients to question doctors about tests  Needing specific knowledge of tests to question doctors  Not being able to questions doctors due to language barrier  Not being able to questions doctors due to neurodivergence  Not questioning doctors about tests  Not questioning doctors about tests due to lack of understanding about rationale  Not questioning doctors due to different abilities  Older patients not questioning tests  Opportunity to ask questions being an important aspect of ED care  Questioning doctors about how patient care is delivered  Questioning doctors about tests being confrontational for patients  Questioning doctors about tests being influenced by patients' age  Questioning doctors about tests depending on information provided  Questioning doctors about tests depends on individual patients  Questioning doctors about tests influenced by patient life experience  Questioning doctors only if poor treatment  Questioning tests that are not medically indicated  Questioning the utility of repetitive tests without answers |
|  |  | Conflict | Being comfortable disagreeing with doctors about tests  Being comfortable escalating concerns if disagreements with doctors about tests  Being comfortable refusing a test recommended by a doctor  Challenging to navigate conflict with doctors about tests  Doctors communicating the rationale in layman language to resolve disagreements  Doctors not liking patients asking questions  Hesitating to confront doctors strongly  Not refusing a test recommended by a doctor  Not disagreeing with doctors about tests due to their expertise  Patient expectations of tests not aligned to symptoms  Relating a conflict between staff due to patient ID error  Resolving conflict with explicit and detailed information about tests  Resolving conflicts through conversations about test options, risk and benefits  Resolving disagreements by listening to patients  Strongly advocating for tests if clinician-patient disagreement |
|  | Risk-benefits | | Accepting of additional blood tests as long as no additional pain or discomfort from needles  Appreciating the current process of risk-benefit discussion about imaging tests  Being scared by the unanticipated physical effects during imaging tests  Being surprised at unanticipated physical effects during tests  Being thankful for tests facilitating diagnosis  Challenging to have a uniform approach test risks-benefits discussion  Choosing to engage with information about test risk-benefits  Communicating risk-benefits to patients in a simple and easy to understand manner  Communication of test risk-benefits by a staff member  Disagreeing about suitable staff to discuss risks-benefits  Discussing risks and benefits of tests before tests  ED staff causing procedural pain  Emphasising discussion of risk-benefits of tests as duty of care  Emphasising that anxiety triggered by tests can be worse than pain  Feeling that mild pain, inconvenience, discomfort, embarrassment are minor risks of tests  Information videos about test risk-benefits in waiting room having downsides  Knowledge of test risk-benefits enabling patient decision making  Needing a risk-benefit discussion about imaging tests  Needing informed consent for tests with risks  Needing intact cognition to have a discussion about risk-benefits  Not having fear of tests due to prior experience  Not having time to discuss risks-benefits  Not needing a risk-benefit discussion about urine tests  Not needing a risk-benefit discussion about blood tests  Not wanting to know risks and benefits of tests  Passively communicating risk-benefits of possible tests for waiting room patients  Patient anxiety about painful procedures being related to previous experiences  Patients having the final decision about tests following a risk-benefit discussion  Patients needing communication and education about risk-benefits  Patients refusing tests despite discussion of risks-benefits  Patients using different strategies to navigate pain and discomfort of tests  Preferring short term pain of tests which will help in the long term  Preferring to have a test done if risks are minor  Priming patients to discussion of risk-benefits using analog or digital aids  Priming patients to risk-benefit discussion through QR codes  Priming patients to risk-benefit discussion through downloadable videos  Recurrent ED visits being detrimental to physical and mental health  Risk-benefit discussion allowing patients to be prepared for pain and discomfort  Risk-benefit discussion allowing patients to overcome the fear of unknown  Risk-benefit discussion being about promoting patient confidence and comfort in best possible care  Risk-benefit discussions depending on the criticality of patient illness  Risk-benefit discussions should be for tests that are clinically indicated  Risk-benefits discussions not being possible in emergencies  Risk-benefits of tests being a delicate topic requiring skilled communication  Tailoring risk-benefit discussion based on lived experience of patients  Tailoring risk-benefit discussion to type of test  The manner of risk-benefit discussion being important  Using aids to prime patients about test risk-benefits being efficient  Using audio communication about test risk-benefits  Using multimodal approach for test risk-benefit discussion  Using visual communication about test risk-benefits  Varying patient risk tolerance to different painful procedures  Wanting simple information about risks and benefits of imaging tests  Wanting to know details of risk-benefits of tests  Wanting to know the risks of tests if risks are signficant |
| Therapeutic relationship | Empathy | Empathy for patients | Appreciating empathetic care by ED staff  Appreciating empathetic care for complex patients from ED doctors  Appreciating generational shift of doctors towards being more sympathetic  Considering patient comfort during tests  ED staff not taking patient symptoms seriously  ED staff showing variable empathy towards patients  Empathetic approach of ED staff precluding the need for an independent patient advocate  Empathy at triage facilitating care in ED  Empathy being an important trait for medical professionals  Patient being taken seriously by ED staff after vitals sign measurement  Patients prioritising personal illness  Perceiving lack of empathy when not taken seriously  Perception of ED staff dependent on empathetic communication  Putting up with pain in waiting room knowing there are sicker patients  Rudeness of ED triage staff increasing the risk of patient aggression  Varying degrees of empathy of patients about fellow patients' needs |
|  |  | Empathy for staff | Acknowledging ED staff are busy  Acknowledging ED staff working in a information limited setting  Being concerned about ED staff burnout due to isolated individual advocacy  Complaints about individual ED staff for systemic issues being unkind  Feeling dreadful for ED staff not coping due to busyness and staff shortage  Feeling sad about ED staff perception of being underappreciated  Feeling that complaining could jeopardize ED staff jobs  Feeling that ED staff need frequent check-ins about wellbeing and breaks  Feeling that ED staff need to have breaks for self-care  High stress environment leading to ED staff burnout  Not complaining about unkind ED staff behaviour  Not complaining due to empathy about ED staff working conditions  Patients advocating for reducing case loads of busy ED staff  Patients being aware of the high work loads of busy ED staff  Patient reported experience not being accurate due to empathy towards stressed staff  Supporting ED staff in dealing with trauma from negative experiences  Understanding that time constraints of ED staff impact care |
|  | Trust | | Community trusting healthcare professionals to look after them and make them better  Knowledge of test rationale instilling trust in ED care  Trusting ED staff to do the right tests and provide right care  Trusting ED staff to manage risks of tests |
|  | Respect | | ED triage staff being rude  Feeling disrespected due to negative ED experience  Feeling worthless and disrespected when concerns are dismissed  Respecting staff who like to learn about patient illness  Treating patients with respect |
|  | Rapport | | Good rapport enabling clinician-patient conversation about appropriate ED tests  Good rapport enabling education of community via patients  Investing time to build rapport enabling good patient care  Listening being critical about building rapport  Non-judgmental, non-stigmatising, non-discriminatory approach being essential for rapport |
| Quality of care |  | | Appreciating that staff training impacts quality of ED care  Being concerned about lack of confidentiality of medical records due to routine processes  Being concerned about the lack of privacy in waiting room  Being embarrassed by the lack of privacy at ED triage  Being concerned about privacy breach from computer screens  Ensuring appropriate patient follow-up for non-emergency care  Experiencing embarrassment to talk at triage due to lack of privacy  Noting lack of infection control practices in ED waiting room  Needing multidisciplinary specialists to ensure optimal care  Pedantic documentation enabling more experienced clinicians consider alternatives  Providing feedback about suboptimal care being a patient responsibility  Quality of care at ED triage impacting care in the rest of ED journey  Questioning patients whilst under the influence of medications  Repetitive questioning of narcotised patients being weird  Research with community enabling better patient care  Thoroughness being an important aspect of ED care |
|  | Timeliness |  | Acknowledging that busyness of ED impacts efficiency of care  Appreciating timely ED care  Appreciating that busyness of ED impacts timeliness of care like analgesia  Appreciating that timely care depends on a combination of factors  Appreciating that timely care depends on availability of skilled staff  Appreciating that timely care depends on equipment availability  Feeling that better time management by staff can improve ED care |
|  |  | Waiting time or Waiting room | Acknowledging ED wait due to non-critical illness  Addressing non-medical needs of waiting patients being important  Appreciating quality of ED care despite long waiting time  Appreciating separate waiting area for children  Appreciating short wait time despite non-critical illness  Being angry at prolonged ED wait time for a minor injury  Being frustrated at prolonged wait times whilst being aware of high workload  Children being anxious or upset due to prolonged wait in a high stress environment  Collecting details about patient symptoms in the waiting room  Communication about triage being very important for waiting patients  Complaining about wait time  Early tests before medical assessment reducing patient waiting time  Feeling bad about complaining about waiting time  Good communication about rationale and wait time for tests improving patient outcomes  Good communication about wait time for test results calming patients  Good communication about wait time for test results enabling patients to make plans  Highlighting the absence of a safe waiting area for patients discharged in the early hours of the day  Information from patients in waiting room assisting decisions about tests  Needing to tailor waiting room primer strategies to patient abilities  Noticing behaviourally disturbed patients in waiting room  Noticing the lack of vital sign performance in ED waiting room  Noticing patients with preventable diseases in waiting room  Open communication about reasons for delayed care provision  Patients being frustrated with prolonged waits for test results in ED Short Stay  Prolonged wait due to miscommunication in waiting room  Prolonged wait for ED discharge due to delay in decision making  Television may calm patients waiting for test results in short stay  Variability in efforts of ED triage staff in locating waiting patients  Waiting for a long time after being called back by ED staff  Waiting for a long time on a second ED presentation for the same problem  Waiting time being an issue for patients  Waiting room patients being a captive audience for risk-benefit primer |
|  | Resources | | Acknowledging that unnecessary tests can waste resources  Acknowledging tests being done due to lack of ED space and staff  Appreciating the rational use of resources for diagnostic testing  Being aware of the cost of unnecessary tests  Being uncomfortable as a caregiver due to lack of ED resources  Cost of tests being offset by prevention of adverse events from timely diagnoses  Feeling that early tests help manage resources  Feeling that early tests would be costly  Feeling that ED lacks adequate resources and staff  Healthcare resources being finite  Inadequate resources necessitating prioritisation of ED care  Inappropriate ED visits resulting in wasted resources  Increasing demand for ED services without a proportionate increase in resource allocation |
|  | Equity | | Deficits in emergency care existing for all patients  ED care needing to be consistent for all patients  Feeling misallocation of resources due to avoidable ED presentation  Supporting tests if equitable resource allocation |
|  | Safety | | Being concerned about personal safety due to other ED patients in custody  Feeling that early tests facilitate patient safety  Feeling unsafe due to negative ED experience  Patients being concerned about safety in ED waiting room  Parents being concerned about safety of children in waiting room due to agitated patients  Patients getting anxious in the high stress ED environment  Questioning non-performance of tests despite clinical deterioration  Questioning the lack of accountability for adverse patient outcomes  Questioning the lack of accountability in not performing tests despite clinical deterioration  Waiting room being an area of uncertainty and anxiety for patients  Waiting room being a high stress area for patients due to behavioural disturbances  Wondering about ED redesign to cohort behaviourally disturbed patients |
|  | Complexity |  | Access block complicating ED care  Acknowledging the complexity of care provision by ED staff  Care prioritisation for some patients delaying care for others  Emphasising the need for better patient flow to enable ED care for emergencies  Navigating the ED easier for patients familiar with hospital processes  Questioning the possibility of an on call person to resolve complex legal situations |
|  |  | Fallibility | Acknowledging some patients may expect perfection  Acknowledging that media influences the perception that ED staff should not miss diagnoses  Agreeing that the community does expect ED staff not to miss diagnoses  Being confident in care with effective communication even if missed diagnoses  Change over and experience of ED staff influencing missed diagnoses  Complaints driving ED staff perception that patients expect perfection  Complexity of ED care resulting in error  Disagreeing that community expects ED staff to be right every time  ED staff being human  Expectation of perfection being from multidisciplinary specialist team rather than ED alone  Expectation of perfection from ED staff not being realistic  Expecting perfection from ED staff  Feeling sorry for staff weighed down by perceived expectation of perfection  Feeling that community does expect ED staff to not miss diagnoses in children  Feeling that missed diagnoses are best handled with honest conversations about root causes  Insufficient knowledge about complex patients influencing missed diagnoses  Not expecting perfection from ED staff  Patient concern for self influencing expectation of perfection in ED  Rapidly evolving knowledge base influences missed diagnoses |
| Patient-centred care | Meaning and Importance | | Contrasting patient-centred and generic approaches to testing  Good communication being an important part of patient centred care  Person-centred care being about ED staff taking time out and listening |
|  | Advocacy | Elements of advocacy | Advocacy being about calling for attention if clinical deterioration  Advocacy being about helping patients who are vulnerable and cannot self-advocate  Advocacy being about keeping the patient informed about what is happening  Advocacy being about making patients aware of ED processes  Advocacy being about empathy and making patients feel heard |
|  |  | External  advocacy | Advocacy being important for first time ED patients  Advocacy being important for unaccompanied patients  Advocating for ED clients despite prior negative ED experience  Advocating for elderly First Nations patients by questioning inconsistent capacity assessment  Advocating for fellow patients  Advocating for First Nations patient care with doctors  Appreciating advocacy by ED staff  Feeling ED staff are too busy to be advocating for patients  Feeling that all First Nations patients need advocacy due to systemic gaps, judgement and stigma  Feeling that ED staff need to advocate for patients without capacity if no other decision maker  Not preferring a patient advocate  Offering a patient advocate for all patients at triage  Patients' family advocating for tests by questioning doctors  Presence of dedicated patient advocate helping patients  Presence of dedicated advocate logistically challenging due to patient volume  Strong advocacy enabling patients get optimal care  Tailoring the need for patient advocate to individual patients |
|  |  | Self  advocacy | Advocating for communication by ED staff in layperson language  Calm and rational manner enabling self- advocacy  Connection with others with shared life experiences enabling self-advocacy  Feeling challenged during self-advocacy  Feeling confronted when self-advocating for optimal ED care  Knowledge about illness and care enabling self-advocacy  Needing persistent self-advocacy  Needing self-advocacy despite blood tests  Needing self-advocacy to ensure optimal care in ED  Needing strong advocacy to ensure optimal ED care  Not getting tests or answers despite self-advocacy  Patients expressing frank opinions about their care with or without diplomacy  Patients needing strong self-advocacy to be taken seriously by ED staff  Patients not knowing self-advocacy  Patients with chronic conditions being experts about their bodies response to illness  Persistent self-advocacy leading to an optimal outcome  Self-advocacy being a difficult skill to learn  Self-advocacy being a learnt skill  Self-advocacy being enabled by rational calm despite internal anxiety  Self-advocacy by disagreeing with doctors about care without anger or abuse  Self-advocacy of tests not recommended by doctors being a challenge  Self-advocacy of tests not recommended by doctors needing communication skills  Self-advocacy of tests not recommended by doctors needing confidence  Self-advocacy whilst disagreeing with doctor being a challenge  Self-advocacy without anger being a subtle skill  Strongly advocating for self about tests  Strongly advocating for patients having detailed knowledge of illness  Strong self-advocacy enabling conflict resolution about tests |
|  | Assessment | | Accepting tests before medical assessment if indicated based on clinical presentation  Considering patient benefits of tests prior to tests  Early testing before medical assessment being efficient  Feeling that early pre-assessment tests may help triage patients  Feeling that negative results of pre-assessment tests serve as a baseline for future  Feeling that test before medical assessment would free up bed space  Questioning tests ordered without medical assessment  Supporting performance of tests before medical assessment  Supporting tests targeted to symptoms before medical assessment  Wanting clear communication about tests done prior to medical assessment  Wanting to know rationale of tests performed prior to medical assessment |
|  | Engagement | | Adapting the performance of blood tests in needle-phobic patients  Appreciating being part of research focussed on community expectations  Being thankful for the opportunity to contribute to care improvement  Clarifying whether patients can eat enabling better care  Communication facilitating patient-centred decision making about tests  Emphasising patients right to know all aspects of care  Emphasising the importance of patient education in improving community health literacy  Hoping research changes culture to prevent patient adverse outcomes  Needing to know patient values to individualise tests  Patients having the final choice after a conversation about tests  Respecting patients’ decision about tests after a detailed conversation  Rudeness of triage staff impacting patient engagement in care  Tailoring communication about tests to individual patients’ cognitive status  Tailoring communication to individual patients being a challenge  Tailoring discussion about tests to individual patient abilities  Wanting ED staff to gauge the level of patient understanding prior to communication about tests  Wanting to know test rationale of tests as this is about patients’ health  Wanting to know the results of tests as this is about patients’ health |
|  | Experience | Positive | Patients with chronic conditions being used to tests  Positive ED experiences outweighing isolated negative experience  Rapid ED care exceeding patient expectations |
|  |  | Negative | Adverse outcome occurring when patients not taken seriously  Being annoyed by having to repeat their illness story to multiple doctors  Being confronted with ED visit due to prior negative experience  Being dissatisfied with incident investigation of an adverse outcome  Being dissatisfied with the dismissive attitude of ED staff  Being frustrated by ED staff not acknowledging seriousness of symptoms  Being horrified at the unkind behaviour of ED staff  Being respectful of ED staff despite prior negative experience  Being stressed by unkind ED staff behaviour  Being upset by mislabelling of patients with an illness  Deciding not to seek healthcare due to negative experience at a care facility  Feeling disappointed about not getting ED care for an urgent, easily fixable problem  Feeling forgotten by ED staff due to delay in promised care  Feeling hurt due to negative ED experience  Feeling upset at not being treated adequately for severe pain  Having positive and negative ED experiences  Needing a support person to ensure appropriate ED care  Negative experience due to not having a test  Negative healthcare experience leading to major patient life event  Not being given any analgesia in ED waiting room  Not making the right diagnosis despite tests  Patients complaining despite best ED care  Patients hearing ED staff complain about other ED staff  Patients seeking tests privately if not getting the test in ED  Patients sharing negative ED experiences with other community members  Relating negative ED experience with difficult prolonged painful procedure  Relating negative experience of ED care  Relating negative experience of ED care due to poor decision making  Relating negative experience of ED care due to poor time management  Relating negative experience with non-ED care |
|  | Vulnerability |  | Anxiety not being helped by relaxation techniques  Anxiety not being helped by relaxation techniques  Being frustrated at the challenges of decision-making about patients without capacity  Being shocked by the number of police in ED  Cognitively impaired patients being unable to have conversations about tests  ED patients needing reassurance to overcome fear of unknown  ED visit inducing anxiety in older indigenous patients due to fear of death  Helping busy ED staff in caring for patients with special needs  Noticing other sick patients inside ED  Noticing the presence of patients in police custody  Noticing the use of time, staff and resources in caring for patients in custody  Patient with cognitive impairment being unable to explain reason for ED visit  Reviewing medical records may help rationalise testing in vulnerable patient cohorts  Routine tests might sometime help vulnerable patients who do not engage with health systems  Substitute decision making about tests for patients who are not competent  Worsening anxiety with worsening physical symptoms  Unconscious patients being unable to provide a history to inform tests |
|  |  | Cultural safety | Appreciating the cultural appropriateness of ED staff  Being questioned about indigenous identity due to appearance  Being questioned about the need to see an indigenous support person  Cultural safety being about building rapport without judgement and stigma  Culturally safe environment enabling First Nations patients to speak up and escalate concerns  Deficits in emergency care being more pronounced for First Nations patients  Emphasising the need to individualise care by listening to First Nations patients  Feeling culturally safe and appropriate when supported by indigenous liaison worker  First Nations patients feeling stigma and judgement due to language barrier  First Nations patients feeling stigma and judgement due to language barrier  First nations patients needing cultural safety to engage with ED mental health clinicians  First Nations patients scared of speaking up due to concerns about discrimination  Highlighting dedicated services for First Nations patients who did not wait for ED care  Indigenous liaison officers assisting First Nations patients with language barriers  Indigenous liaison officers facilitating better clinician-patient communication  Indigenous liaison officers instilling confidence in First Nations patients to speak up  More First Nations staff enabling cultural safety  Navigating gender specific needs of First Nations patients with open communication  Offering Older First Nations patients a choice of gender specific care  Reading indigenous liaison officer notes helping medical staff to know patient better  Respecting gender specific needs of First Nations patients enabling cultural safety  Visual aids enabling better communication with older first nations patients  Worsening health of First Nations patients who do not seek timely ED care |
|  |  | Power imbalance | Accepting doctors' recommendation without questioning  Always agreeing with tests recommended by a doctor  Deferring to the expertise of healthcare professionals about tests  Junior clinicians being powerless to overrule senior decision making about tests  Listening to doctors as disagreements can be difficult to handle  Not advocating or speaking up in disagreement about care  Not complaining about procedural pain  Not disagreeing with doctors about tests due to their expertise  Not disagreeing with doctors advice about tests despite concerns  Persistent concerns after ED visit prompting GP consult for tests |
|  |  | Health literacy | Adapting conversations about tests to patient knowledge  Describing illness in varying detail  Health literate patients being able to better understand test discussion  Not interested in knowing all the details about care  Online research influencing patients expectations of tests  Patients doing research to know more about their care  Patients not being aware of ED patient volume and acuity  Patients using internet to learn about the cause of their illness  Patients with health background having prior knowledge of types of tests  Written passive communication not helping patients who cannot read |
| Seeking ED care | ED attendance |  | Being aware that ED is for emergency care  Being compelled to visit ED due to worsening chronic curable illness  Feeling uncomfortable presenting to ED with a non-urgent problem  Insufficient community awareness of appropriate ED visit reasons  ED substitutive care providers having limited facilities  Presenting to ED after being referred by ED substitutive care providers  Presenting to ED as unable to pay for primary care provider  Presenting to ED due to difficulty in timely GP consultation  Presenting to ED to avoid worsening clinical condition  RACF’s sending patients to ED for tests due to legal reasons  Visiting ED despite being aware of systemic issues and ED appropriateness |
|  | ED avoidance |  | Avoiding ED because ED staff are really busy  Avoiding ED presentation due to anticipated waiting times  Avoiding ED presentation due to concerns about being recognised by work colleagues  Avoiding ED presentation due to due to timely non-ED specialist intervention  Avoiding ED presentation to not waste resources  Being sad about ED avoidance due to resource constraints  Highlighting avoidable ED presentation due to chronic treatable problem  Lack of transport options limiting ED visits by First Nations patients  Not wanting unnecessary transport to ED for testing  Patients not waiting for care to not burden ED  Patients with chronic illness needing support to avoid ED presentations  Seeking alternative healthcare providers to avoid ED presentation |
|  | Appreciation |  | Appreciating the quality of ED care  Appreciating quality of ED care despite resource constraints |
